# Are gifted adolescents more satisfied with their lives than their non-gifted peers?

**DOI:** 10.3389/fpsyg.2015.01623

**Published:** 2015-10-20

**Authors:** Sebastian Bergold, Linda Wirthwein, Detlef H. Rost, Ricarda Steinmayr

**Affiliations:** ^1^Department of Psychology, Technical University of DortmundDortmund, Germany; ^2^Faculty of Psychology, Southwest University ChongqingChongqing, China; ^3^Department of Psychology, Philipps-Universität MarburgMarburg, Germany

**Keywords:** life satisfaction, giftedness, subjective well-being, intelligence, adolescents, gender differences

## Abstract

Studies investigating the life satisfaction of intellectually gifted and non-gifted students are scarce and often suffer from methodological shortcomings. We examined the life satisfaction of gifted and non-gifted adolescents using a rather unselected sample of *N* = 655 German high-school students (*n* = 75 gifted), adequate comparison groups of non-gifted students, and a clear definition of giftedness (general intelligence *g* > 2 SD above the mean). There was no difference in life satisfaction between gifted and non-gifted adolescents (*d* < |0.1|). Girls reported somewhat lower life satisfaction scores than boys (*d* = 0.24). However, this result was not specific to giftedness but was instead found across the entire sample. Thus, gifted girls were not found to be especially unsatisfied with their lives. Our findings support previous research showing that giftedness is not a risk factor for impaired psycho-social well-being of boys or girls.

## Introduction

In recent decades, there has been a growing interest in the questions of whether certain groups of individuals are more satisfied with their lives than others and which individual determinants contribute to the subjective well-being of an individual (e.g., Eid and Larsen, 2008). Research has shown that individual characteristics (e.g., personality variables; see Lucas, 2008) contribute to a person’s life satisfaction and well-being. Diener (1984, p. 559) supposed that “intelligence is a personality variable that would be expected to relate strongly to subjective well-being because it is a highly valued resource in this society.” Hence, one could assume that people who score high on intelligence tests—intellectually gifted individuals—might be more satisfied with their lives than non-gifted individuals. However, there is still a tendency to assume that gifted individuals are at higher risk for developing emotional and social difficulties and might report a lower life satisfaction than non-gifted individuals (e.g., Ziegler and Raul, 2000). Empirical research focusing on the life satisfaction of gifted adolescents is scarce (e.g., Neihart, 1999; Jones, 2014), and the few existing studies have been plagued by various methodological shortcomings such as small and/or preselected samples and/or a lack of appropriate comparison groups and/or imprecise definitions and operationalizations of giftedness. Thus, the aim of the current study was to shed some light on the life satisfaction of adolescents by using reliable and valid instruments to assess intellectual giftedness as well as life satisfaction and by comparing a rather unselected group of gifted adolescents with an appropriately matched non-gifted group.

### Defining Intellectual Giftedness and Life Satisfaction

There are several different definitions of “intellectual giftedness” (Sternberg and Davidson, 2005), and many of them advocate a multidimensional approach. For example, some conceptions assign major roles to creativity and motivational constructs and regard giftedness as high achievement rather than as high potential (e.g., Renzulli, 1978; Mönks, 1990). However, there are many theoretical as well as methodological reasons to consider high general intelligence to be the core indicator of giftedness (see Rost, 2013). For example, intelligence is a highly stable construct and the best singular psychological predictor of many important life outcomes such as scholastic achievement, professional success, and socio-economic status. Furthermore, many multidimensional models require high scores on several different constructs, thus posing the problem that the group of gifted individuals will shrink and finally vanish as the number of constructs rises. Also, intelligence can be reliably assessed, and many sound intelligence tests are available, whereas measures of, for example, creativity and motivation often suffer from lower psychometric properties and poor validity in direct comparison with intelligence tests (see Robinson, 2005). Thus, the use of intelligence test scores (IQ > 2 SD above the mean) to define giftedness has prevailed in the empirical literature (Rost, 1993; Roznowski et al., 2000; Wirthwein and Rost, 2011; Baudson and Preckel, 2013).

Comparable to the various definitions of giftedness, there are also a lot of different conceptualizations of well-being (e.g., life satisfaction, happiness, quality of life), and this makes it difficult to compare the results found in different studies. In the current study, we focus on the construct of life satisfaction because it has been investigated more frequently than other well-being variables and is seen as the most stable component of subjective well-being (Suldo et al., 2006). Life satisfaction refers to a “global evaluation by the person of his or her life” (Pavot et al., 1991, p. 150). It has frequently been examined in the context of subjective well-being, including cognitive (i.e., life satisfaction) as well as emotional components (i.e., positive and negative affect; see Diener et al., 1999). Besides judgments of global life satisfaction, satisfaction in different domains (e.g., satisfaction with school, family, friends) can be differentiated (Diener et al., 1999).

In the current study, we rely on global life satisfaction self-reports of gifted and non-gifted adolescents identified via a well-established intelligence test. We focus in particular on gifted and non-gifted adolescents because this age group might be especially susceptible to low life satisfaction (e.g., Gilman and Huebner, 2003).

### Intellectual Giftedness and Life Satisfaction

Terman was the first to study the life satisfaction of intellectually gifted individuals in his outstanding longitudinal study “Genetic studies of genius” (e.g., Sears, 1977), indicating a positive relation between intelligence and life satisfaction. The life satisfaction of gifted individuals was also investigated in the Study of Mathematically Precocious Youth, drawing on large samples. The study found that the gifted individuals (in early and middle adulthood) displayed high levels of life satisfaction (Lubinski et al., 2006, 2014) and high self-esteem (Swiatek and Benbow, 1991). Diener and Fujita (1995) supposed that people with high personal resources (inter alia high intelligence) are better able to achieve their goals and, hence, might be happier than people with low personal resources. Correlational studies conducted with children and adolescents have mainly found negligible associations between intelligence and life satisfaction (e.g., Huebner and Alderman, 1993: *r* = -0.08; Chmiel et al., 2012: *β* = 0.04). Negligible associations have also been found in studies investigating life satisfaction and intelligence in adults (e.g., Watten et al., 1995; Rode et al., 2008). Studies focusing on different affective well-being measures have shown heterogeneous associations with intelligence. For example, Kirkcaldy et al. (2004) analyzed students from different international large scale assessments and found a correlation between happiness and intelligence of *r* = 0.44, whereas Owuchi and Yoshino (1975) reported a correlation of only *r* = 0.07.

There are two different hypotheses about the social and emotional characteristics of the gifted: the harmony hypothesis and the disharmony hypothesis (e.g., Mönks, 1963). According to the harmony hypothesis, gifted individuals show a more balanced personality profile and are, for example, more successful and more socially competent than non-gifted individuals (e.g., Plucker and Callahan, 2008). Hence, according to this hypothesis, gifted individuals should report higher life satisfaction than non-gifted individuals. However, according to the disharmony hypothesis, gifted people should show social and emotional difficulties and a higher risk for developmental disorders compared with the non-gifted (e.g., Neihart, 1999).

Most empirical research has shown support for the harmony hypothesis: differences between gifted and non-gifted individuals across various age groups and various emotional and psycho-social variables are mainly in favor of the gifted or are rather negligible (Czeschlik and Rost, 1988; Rost, 1993, 2009; Plucker and Callahan, 2008). For example, gifted students were found to be somewhat more popular and somewhat less rejected by their classmates than non-gifted students (Rost and Czeschlik, 1994a; Czeschlik and Rost, 1995). In a study by Rost and Czeschlik (1994b), gifted and non-gifted 10-year-olds did not differ in psycho-social adjustment, namely anxiety, extraversion, diffidence, sociability, problem behavior, or social contacts, as indicated by their parents, their teachers, and the children themselves. Results were largely the same for adolescents: there were no differences between gifted and non-gifted adolescents in social integration, emotional state, or number of friends. If anything, gifted adolescents had somewhat less contact with their friends but were, however, not socially isolated (Schilling, 2002; Rost, 2009). Furthermore, they had a high level of confidence in their cognitive abilities and a low level of fear of failure (Neitzke and Röhr-Sendlmeier, 1992; Schütz, 2009). Results supporting the disharmony hypothesis have mainly been due to the methodological shortcomings of the studies such as the use of preselected and very small samples (e.g., from counseling centers or by using students who attend special gifted courses), often in combination with the lack of an adequate non-gifted control group. Moreover, gifted individuals are frequently not identified via reliable and valid intelligence tests but instead via teacher, peer, or parent nominations (see Rost, 1990, 2009), and the definitions of giftedness vary considerably across different studies.

Taking into account those studies that have employed a non-gifted control group, gifted individuals do not differ from non-gifted groups in mental health or various well-being measures (e.g., Zeidner and Shani-Zinovich, 2011; Jones, 2014). But only a few studies have explicitly examined the life satisfaction (i.e., the cognitive component of subjective well-being) of gifted compared with non-gifted adolescents. Ash and Huebner (1998) compared *n* = 61 gifted middle school students with *n* = 61 non-gifted students. They found an effect size of *d* = 0.24 in favor of the gifted. Shaunessy et al. (2006) compared *n* = 33 gifted adolescents with *n* = 179 other students from the same school. The effect was not statistically significant and very small (*d* = 0.14 in favor of the gifted). In a study by Loeb and Jay (1987), effect sizes ranging from *d* = 0.03 to *d* = 0.50 (again in favor of the gifted) were detected in *n* = 125 gifted and *n* = 102 non-gifted fourth to sixth graders across different well-being measures (but not explicitly life satisfaction). Other studies focusing on different well-being measures in children and adolescents (Chapman and McAlpine, 1988; Jin and Moon, 2006) have shown comparable results. Although the aforementioned studies included comparison groups, they still had some methodological shortcomings: for example, the study by Ash and Huebner (1998) used teacher recommendations, grades, and student interviews to identify gifted students, and Shaunessy et al. (2006) also used teacher nominations and grades besides intelligence test scores; in addition, the gifted students were enrolled in a special course for gifted and talented students. However, the results found for adolescents were comparable to the results found for gifted adults in a methodologically sound study by Wirthwein and Rost (2011). In this study, intelligence test scores were exclusively used to identify intellectually gifted and non-gifted adults, and the gifted individuals were not preselected via teacher nominations or parents. Moreover, the control group was matched on age, gender, and socio-economic status. The authors found no statistically significant difference between the two groups on global life satisfaction as well (*d* = 0.16 in favor of the gifted).

Several other studies have been conducted on gifted adolescents, mainly focusing on variables such as self-concept or other measures of mental health (see Martin et al., 2010; Zeidner and Shani-Zinovich, 2011; Jones, 2014). These studies also found only small or negligible differences between the investigated groups, again supporting the harmony hypothesis.

Some researchers have argued that gifted girls compared with gifted boys are at a particularly higher risk for developing emotional or social difficulties (see Reis, 2004), and hence, gifted girls might report lower life satisfaction. This research topic has received little attention so far. To our knowledge, only Wirthwein and Rost (2011) investigated gender differences in life satisfaction between gifted and non-gifted adults. The authors did not find a giftedness × gender interaction of statistical or practical significance (η^2^ = 0.01). We are not aware of any study that has focused on gender differences in gifted adolescents’ life satisfaction while also taking into account non-gifted adolescents’ life satisfaction.

### The Present Study

The aim of the present study was to shed some light on the life satisfaction of gifted compared with non-gifted adolescents. As already mentioned, previous studies have suffered from several methodological shortcomings such as imprecise definitions of giftedness, lack of an appropriately matched non-gifted control group, or small preselected samples. With our study, we tried to avoid some of these shortcomings. Therefore, we analyzed the data of a relatively unselected sample, and we used the results from a well-established intelligence test as the criterion to define intellectual giftedness (see Robinson, 2005; Rost, 2009). Moreover, we analyzed a comparable group of non-gifted adolescents. With regard to the few methodologically sound existing studies that have focused on the life satisfaction of gifted individuals, we expected that gifted individuals would report similar or higher life satisfaction scores than non-gifted individuals (Ash and Huebner, 1998; Shaunessy et al., 2006; Wirthwein and Rost, 2011).

Another aim was to investigate whether gifted girls might display lower life satisfaction scores than gifted boys while also taking into account the life satisfaction scores of non-gifted girls and non-gifted boys. To our knowledge, this is the first study to investigate this research topic in a sample of adolescents. Thus, we did not formulate specific hypotheses concerning this topic.

## Materials and Methods

### Participants

The sample comprised *N* = 655 students (358 girls) attending a German Gymnasium. Students attended either the 11th or 12th grade and were on average *M* = 16.65 (*SD* = 0.71) years of age. The Gymnasium is the highest track in the German secondary school system and the option most frequently chosen for receiving the Abitur, a school-leaving certificate that is mandatory for university enrollment. After finishing elementary school (4th grade), students are selected for one of several different school tracks. The highest performing students are selected for the Gymnasium (about 30–45% of each cohort, depending on the specific local conditions). Thus, students attending a Gymnasium are high performing and are above average in intelligence (see Steinmayr et al., 2010), and the prevalence of gifted students is higher in Gymnasium samples than in the entire student population. Data from two studies were combined. The first study (see Amelang and Steinmayr, 2006) took place in 2004 and the second one in 2007 and 2008. Participation was voluntary. Written consent was obtained from the parents of the under-aged students. Samples did not differ with regard to the variables of interest in the present study.

### Measures

#### Intelligence

We administered the basic module of the Intelligence-Structure-Test 2000 R (*Intelligenz-Struktur-Test 2000 R*; *IST 2000 R*; Liepmann et al., 2007). This test is based on Thurstone’s and Cattell’s intelligence theories and measures verbal, numerical, and figural reasoning ability. The composite score indicates general reasoning ability, which is closely tied to general intelligence (*g*; see Brand, 1996; Jensen, 1998). The IST 2000 R is one of the most renowned and often applied intelligence tests in the German-speaking countries. Its psychometric properties and validity are well-established (e.g., Bühner et al., 2006; also see Schmidt-Atzert and Rauch, 2008). It is Z-scaled with a mean standardized intelligence score (SIS) of *M* = 100 and *SD* = 10.

Following Terman et al. (1925) and in accordance with other studies (e.g., Rost, 1990, 1993, 2009; Roznowski et al., 2000; Thompson and Oehlert, 2010), we defined giftedness as a high level of Spearman’s (1904, 1927) general intelligence *g*, most often indicated by means of an intelligence score that is more than 2 SD above the mean. Applied to the intelligence test we used, we diagnosed giftedness if a student’s SIS was higher than 120 (i.e., if a student belonged to the upper 2 percent of the population). Accordingly, on the basis of their SIS, students were categorized into four different groups. The below-average (SIS ≤ 90) group comprised *n* = 8 students (7 girls; *M*_age_ = 17.25 years, *SD*_age_ = 1.49). An average SIS (90 < SIS ≤ 110) was displayed by *n* = 460 students (286 girls; *M*_age_ = 16.66 years, *SD*_age_ = 0.70), and *n* = 112 students (46 girls; *M*_age_ = 16.58 years, *SD*_age_ = 0.67) had above-average intelligence scores (111 ≥ SIS ≤ 120). A subsample of *n* = 75 students (19 girls; *M*_age_ = 16.61 years, *SD*_age_ = 0.72) was categorized as gifted (SIS > 120). For propensity score matching (PSM; see below), we used only the average SIS group as the comparison group.

#### Life Satisfaction

Life satisfaction was measured with the *General Life Satisfaction Scale* developed by Dalbert (2003). It consists of seven items (e.g., “I am satisfied with my life,” “I consider myself a happy person”) that can be answered on a 7-point scale (1 = “does not apply to me at all” to 7 = “fully applies to me”). Negatively worded items were recoded so that high scores indicated high satisfaction with life. The scale measures the cognitive dimension of subjective well-being. It describes satisfaction with one’s present and past life and with one’s perspective on the future. In our study, the internal consistency (Cronbach’s α) of the General Life Satisfaction Scale was excellent (α = 0.93 for the groups of gifted and non-gifted students).

#### Sociodemographic Data

All students reported their gender, age, and parents’ highest school-leaving qualifications. The following categories were formed for the parents’ qualifications: 0 = no graduation, 1 = lower secondary education (Hauptschulabschluss), 2 = secon dary school certificate (Mittlere Reife), 3 = entrance qualifi cation for university of applied sciences (Fachhochschulreife), 4 = Abitur. As an index of parental educational level, we used the sum of the highest school-leaving qualifications of both parents. Mothers’ and fathers’ educational levels were substantially correlated (*r* = 0.41, *p* < 0.001). The possible range of the composite score was 0–8.

### Procedure

Testing sessions took place at school during a regular school day and were conducted in groups of about 20 students. Trained university students and research assistants administered the tests according to standardized instructions. Participation was voluntary, and students were allowed to take part only if their parents had completed written consent forms. All but five parents agreed. The participation rate in both student groups was about 90%: students who were not present when testing took place were absent due to illness or for other reasons that were not related to our investigation.

### Analyses

#### Propensity Score Matching

In order to generate equivalent groups of gifted and non-gifted students, we used PSM (Rosenbaum and Rubin, 1983). PSM has received increasing attention in the social sciences in recent years as it is a valuable tool for establishing comparability between research groups. To calculate propensity scores, we used a logistic regression analysis with giftedness (0 = non-gifted, 1 = gifted) as the dependent variable and the covariates age, gender (0 = male, 1 = female), and parental educational level as predictors (method: inclusion) (Stuart, 2010). The matching procedure can be performed in several ways (see Thoemmes and Kim, 2011). For our analyses, we used 1:1 nearest neighbor matching without replacement. To ensure the quality of the matching result, we chose a relatively strict caliper of *c* = 0.1 SD of the logit of the propensity score.

After matching, we analyzed differences in the means and variances of the covariates between the groups to test whether age, gender, and parental educational level were actually balanced between the groups (balance property). For this purpose, we used the overall balance χ^2^ test (Hansen and Bowers, 2008) as well as the *L*_1_ statistic (e.g., Iacus et al., 2012). If good balance is achieved, the χ^2^ should be non-significant, and *L*_1_ should be smaller for the matched samples than for the unmatched samples (Iacus et al., 2012). In addition, we inspected univariate statistics for the covariates. Differences in both the means and variances of the covariates should be close to 0. The significance level was set at 5%. We relinquished adjusting the α level in order to keep the β error small. As our gifted sample (and consequently the matched sample of non-gifted students, due to 1:1 matching) was rather small, we additionally calculated Cohen’s *d*. Values of *d* ≥ 0.80 were considered to indicate a large effect, *d* ≥ 0.50 a moderate effect, and *d* ≥ 0.20 a small effect (Cohen, 1988).

Furthermore, we investigated the overlap in the propensity score distributions of the two groups (area of common support). Small areas indicate that effect estimation is restricted to a very specific subsample. By contrast, large areas suggest that the results are representative of the full range of the sample at hand (Thoemmes and Kim, 2011).

#### Group Comparison

After testing for the balance property and inspecting the area of common support, group comparisons were performed. Because the matched nature of the data could affect the estimates of the standard errors and hence the significance level, there is some debate about whether it is permissible in such a situation to use statistics that assume independence of observations (Thoemmes and Kim, 2011). In the present study, we tested the results with corrected (*t*-test for independent samples) and uncorrected (*t*-tests for dependent samples) standard errors. Giftedness (gifted vs. non-gifted) was the independent grouping variable, and the score on the General Life Satisfaction Scale was the dependent variable.

When matching procedures are used, the target group is always compared with a control group that is preselected on certain characteristics and, hence, the generalizability of the results to the rest of the sample can be questioned. Therefore, we also tested whether our findings from the PSM would hold with a relatively unselected comparison group. Since we aimed to control for gender, we used the data that were available from all of the boys (56 gifted and 241 non-gifted) and from the gifted girls (*n* = 19) and drew a random sample of *n* = 82 non-gifted girls out of the group of all students with SIS ≤ 120 (*N* = 580) to achieve parity in the gender distribution between the gifted and non-gifted groups. Then we ran an ANCOVA with giftedness as the independent variable. The gifted adolescents had a higher parental educational level [*t*(396) = -2.51, *p* = 0.01, *d* = 0.25] and were somewhat younger than the non-gifted adolescents [*t*(396) = 0.92, *p* = 0.36, *d* = 0.09]. Since both variables showed (small) correlations with life satisfaction (parental educational level: *r* = -0.07, *p* = 0.16; age: *r* = -0.30, *p* < 0.001), we included them as covariates in the ANCOVA.

To investigate whether gifted girls would display lower life satisfaction scores as compared with gifted boys, non-gifted girls, and non-gifted boys, we ran another ANCOVA on the entire sample, including both giftedness and gender as independent variables. Again, the covariates were age and parental educational level.

#### Missing Data

With the exception of parental educational level, there were no missing data. Regarding parental educational level, the missing data rate was 1.75%. Little’s test indicated that the data were missing completely at random [χ^2^(33) = 29.94, *p* = 0.62]. Nevertheless, to preserve good preconditions for matching, we did not opt for listwise deletion but instead imputed the missing values by means of an expectation maximization algorithm.

## Results

### Propensity Score Matching

**FIGURE 1:**
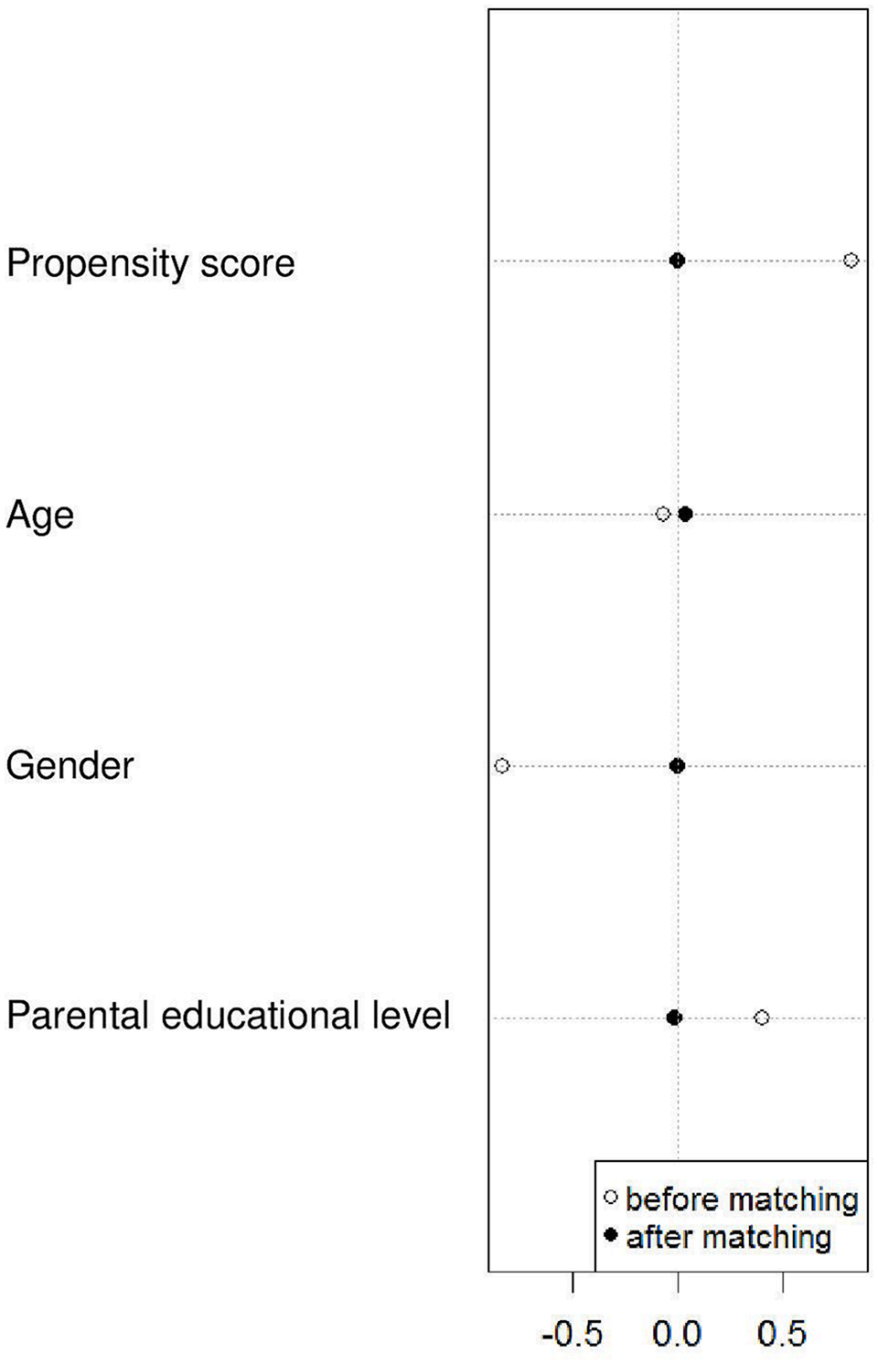
**Standardized mean differences between the gifted and non-gifted students on the covariates before and after 1:1 nearest neighbor caliper (*c* = 0.1) matching without replacement**.

By means of 1:1 nearest neighbor matching without replacement, 74 of the 75 gifted students could be matched. Before testing for group differences, we inspected the quality of the matching results. First, we checked for the balance property. The overall balance test was not statistically significant [χ^2^(3) = 0.06, *p* > 0.99], indicating excellent overall multivariate balance. As required, *L*_1_ was smaller after matching (*L*_1_ = 0.27) than before matching (*L*_1_ = 0.48), suggesting good relative multivariate balance. Univariate analyses of differences in the means and variances of the covariates between the matched gifted and the matched non-gifted samples showed that matching was able to substantially reduce the differences in the means and also some differences in the variances of the covariates (see **Figure 1** and **Table 1**).

**Table 1:** Means (M), standard deviations (SD), and variance ratios (VR) of gifted and non-gifted students on the covariates before and after 1:1 nearest neighbor caliper (*c* = 0.1) matching without replacement.

	Gifted		Non-gifted					
	*M*	*SD*	*M*	*SD*	*VR*	*t*	*p*	*d*
**Before matching**								
Age	16.61	0.72	16.66	0.70	1.05	-0.57	0.57	-0.06
Gender (0 = male, 1 = female)	0.25	0.44	0.62	0.49	0.81	-6.65	<0.01	-0.54
Parental educational level	5.95	1.93	5.17	1.88	1.05	3.30	<0.01	0.29
**After matching**								
Age	16.64	0.69	16.61	0.72	0.92	0.23	0.82	0.04
Gender (0 = male, 1 = female)	0.26	0.44	0.26	0.44	1.00	0.00	>0.99	0.00
Parental educational level	5.92	1.93	5.95	1.87	1.07	-0.09	0.93	-0.01

*Before matching: *N* = 75 (gifted group), *N* = 460 (non-gifted group). After matching: *N* = 74 (both groups). VR = *S*^2^_*gifted*_/*S*^2^_*non-gifted*_. Positive *t*- and *d*-values: differences in favor of the gifted students.*

**FIGURE 2:**
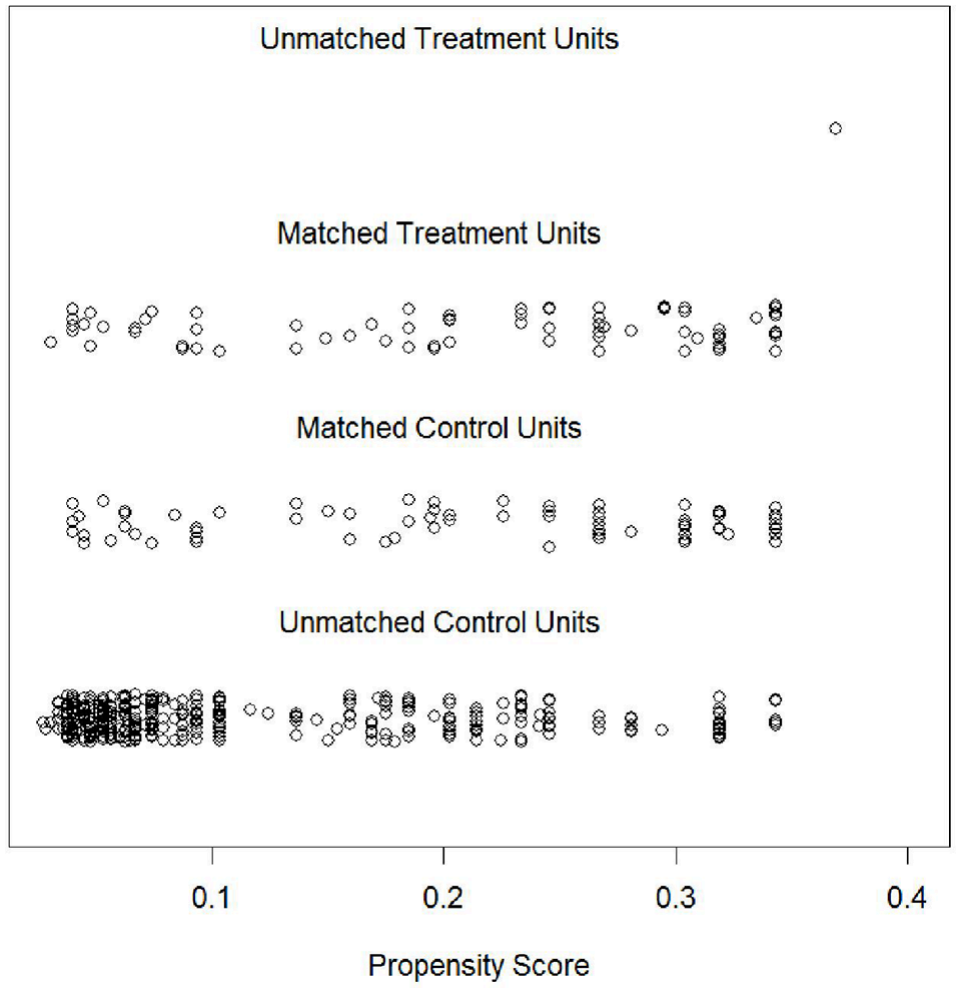
**Distributions of propensity scores for the unmatched and matched gifted (“Treatment”) and non-gifted (“Control”) groups**.

As could already be inferred from the fact that a match was found for all but one gifted student, the area of common support was high. **Figure 2** illustrates the distribution of propensity scores for all matched and unmatched gifted and non-gifted students. As can be seen, only one gifted student’s propensity score was too high to be matched with a student from the non-gifted group. For all other gifted students, suitable counterparts were identified.

Thus, the matching procedure revealed both good balance on age, gender, and parental educational level and a high overlap between the gifted and the non-gifted group so that (1) the two groups were comparable after matching and (2) the results were representative of a substantial array of our sample.

### Group Comparison

After matching, we first computed a *t*-test for independent samples. Life satisfaction was nearly identical between the gifted (*M* = 4.65, *SD* = 1.23) and non-gifted students (*M* = 4.69, *SD* = 1.17). Accordingly, the difference that was found was neither statistically nor practically significant [*t*(146) = -0.20, *p* = 0.84, *d* = -0.03]. We additionally conducted a *t*-test for dependent samples to account for the matched nature of our data. The results did not differ from the previous analysis [*t*(73) = -0.30, *p* = 0.77].

Despite a large area of common support, matched samples are always selected on certain characteristics. Therefore, we also tested whether the finding with a rather selected comparison group would hold when using a relatively unselected comparison group, drawing on a random sample with an adjusted gender distribution (see above). Again, the life satisfaction scores of gifted students (*M* = 4.65, *SD* = 1.22) and non-gifted students (*M* = 4.64, *SD* = 1.15) were virtually the same, and the difference was not statistically or practically significant [*F*(1,394) = 0.08, *p* = 0.78, *d* = 0.03]. Of the covariates, the effect of age [*F*(1,394) = 38.56, *p* < 0.001, η^2^ = 0.089] was statistically and practically significant, with younger students reporting higher life satisfaction. We additionally ran an ANCOVA using the entire sample and controlling for gender. The results were virtually the same [with a significant effect of gender: *F*(1,650) = 17.27, *p* < 0.001, η^2^ = 0.026].

We then included both giftedness and gender as independent variables in another ANCOVA to investigate both the main effect of gender on life satisfaction and the gender × giftedness interaction, using all available student data. Gifted boys displayed the highest life satisfaction scores (*M* = 4.84, *SD* = 1.10), followed by non-gifted boys (*M* = 4.73, *SD* = 1.12), non-gifted girls (*M* = 4.43, *SD* = 1.22), and gifted girls (*M* = 4.11, *SD* = 1.41). The main effect of gender (girls reporting lower life satisfaction than boys) was statistically significant [*F*(1,649) = 9.26, *p* = 0.002, *d* = 0.24]. However, the gender × giftedness interaction was not, and the effect size was negligible [*F*(1,649) = 0.62, *p* = 0.43, η^2^ = 0.001]. The main effect of giftedness was again not statistically significant and negligible in size [*F*(1,649) = 0.46, *p* = 0.50, *d* = -0.05].

## Discussion

So far, there have been only a few studies that have investigated whether intellectually gifted individuals report higher subjective well-being or life satisfaction, respectively, than non-gifted individuals. Even fewer studies have investigated life satisfaction, especially in gifted and non-gifted adolescents, and some of them have been limited by imprecise definitions of giftedness, high selectivity of samples, and a lack of appropriately matched non-gifted comparison groups (see Rost, 2009; Wirthwein and Rost, 2011). Moreover, other studies have frequently used teacher ratings to identify intellectually gifted individuals. In our study, we tried to avoid these methodological shortcomings. Giftedness was clearly defined as a SIS greater than 120 (PR ≥ 98) in general reasoning ability as measured by the IST 2000 R. Regarding life satisfaction, we used a well-established German questionnaire (Dalbert, 2003). As we collected data from regular schools (as opposed to, e.g., special schools or classes for gifted students) and the participation rate was high, our samples could be assumed to display relatively low selectivity with regard to students attending the German Gymnasium. Furthermore, we ensured comparability between the gifted and non-gifted students by means of PSM and tested for the generalizability of our findings.

Our results indicated that there was no difference in life satisfaction between gifted and non-gifted students. This result emerged irrespective of whether we used a selected sample (PSM) versus a random sample or the entire sample (ANCOVA). It was also irrespective of the group comparison method within the PSM-matched samples (correcting vs. not correcting for the matched nature of the data). Although there was a slight variation in both the size and direction of Cohen’s *d* across our analyses, *d* was clearly and consistently below |0.1|, suggesting that life satisfaction does not differ between intellectually gifted and intellectually non-gifted adolescents. This finding was not limited to a comparison with a preselected group of non-gifted students but was rather representative of the entire sample in our study. Our results are in contrast to studies that have suggested that gifted individuals display lower subjective well-being and more socio-emotional problems than non-gifted individuals do (see Neihart, 1999)—often referred to as the disharmony hypothesis (e.g., Mönks, 1963). However, many such studies used highly selective gifted samples. Whenever differences between gifted and non-gifted individuals were found in social, emotional, or other personality variables, there were always sound studies that found no differences between the investigated groups (see Plucker and Callahan, 2008). Hence, it is extremely important to examine unselected samples and make comparisons with appropriate control groups. The results of our study are all the more convincing as our sample was not preselected, and students did not know whether they belonged to the gifted or non-gifted group.

Our findings are in line with other studies that drew on vastly unselected samples and also did not find any differences in subjective well-being or life satisfaction, respectively, between gifted and non-gifted students, even when controlling for confounds such as social and economic background (Ash and Huebner, 1998; Zeidner and Shani-Zinovich, 2011; for adults, see Wirthwein and Rost, 2011). Contrary to the assumptions made by Diener (1984) or Diener and Fujita (1995), intelligence might be a less relevant determinant of life satisfaction. In this context, Wilson (1967) already supposed that intelligence might not be very relevant to life satisfaction. Instead, he emphasized the relevance of personality, coping styles, and other life goals. In sum, as Eid and Larsen (2008, p. 5) stated, “no single condition or characteristic is sufficient to bring about happiness in itself.”

We found that girls reported lower life satisfaction than boys (*d* = 0.24). However, this finding was not specific to giftedness but was instead found across the entire sample. This is closely tied to Wirthwein and Rost’s (2011) finding that there was no significant interaction between giftedness and gender in their exploration of the life satisfaction of adults. Therefore, it can be concluded that intellectually gifted girls are not at higher risk for developing higher emotional or social problems compared with intellectually gifted boys or non-gifted girls (see Reis, 2004). According to most studies on gender differences in life satisfaction, there are only minimal gender differences (see Huebner and Diener, 2008). However, the fact that there was (at least) a small gender difference in life satisfaction in our study justified our matching for gender. The finding that boys reported somewhat higher life satisfaction than girls may be related to gender differences in other variables that might contribute to life satisfaction. Girls have been reported to show lower ability self-perceptions and self-efficacy than boys, to attribute success to external factors, and to attribute failure to low ability (e.g., Nicholls, 1979; Stetsenko et al., 2000; Steinmayr and Spinath, 2009). Furthermore, girls are known to experience more anxiety and have more trouble regulating negative emotions than boys (e.g., Bender et al., 2012). Against this background, slight differences in life satisfaction in favor of boys seem easy to understand.

One might argue that the sample under study was not representative as we recruited students from only the highest track in the German school system. As research indicates that educational level is associated with well-being (Easterbrook et al., 2015), the results we found might apply only to gifted students attending a Gymnasium. However, nearly all gifted students attend a Gymnasium in Germany. In the Marburg Giftedness Project (Rost, 1993, 2009; Wirthwein and Rost, 2011), 151 students out of *N* = 7023 primary school students (third graders) were identified as being gifted, of which *n* = 136 students later on attended a Gymnasium (Sparfeldt et al., 2006). Thus, about 90% of all German gifted students attend a Gymnasium. On the basis of this fact, it makes sense that the likelihood of identifying gifted students at a Gymnasium is very high. This is reflected by the fact that the percentage of gifted students in our sample was markedly higher than one might expect in a representative sample (11.5% compared with 2%). Thus, the Gymnasium is the type of school typically attended by gifted students in Germany. Therefore, the fact that we investigated only students attending a Gymnasium might not have influenced the results as much as may have been the case in other studies of life satisfaction that focused on independent variables other than giftedness.

However, not only the gifted but also the non-gifted students attending a Gymnasiums are a selected group and therefore more intelligent in comparison with students who do not attend a Gymnasium. Therefore, one might suppose that if we had used a non-Gymnasium comparison group, the gifted might have reported lower life satisfaction than the control group because being gifted would have been more salient and might therefore have provided a risk factor for being less socially accepted or integrated. However, studies have shown that gifted students are most often well integrated even in vastly unselected samples (e.g., elementary school children; Rost and Czeschlik, 1994a,b; Czeschlik and Rost, 1995). Furthermore, studies investigating the big-fish-little-pond-effect have provided support for the hypothesis that a comparison with classmates with a low ability level is even beneficial for the academic self-evaluation of gifted individuals (e.g., Zeidner and Schleyer, 1999; Preckel and Brüll, 2010; Arens and Watermann, 2015). And satisfaction with school is, in turn, significantly linked to general life satisfaction, especially among gifted students (Ash and Huebner, 1998).

Another potentially problematic aspect of our sample may be the underrepresentation of underachievers. As students attending a Gymnasium are highly selected on the basis of their scholastic achievement at the end of elementary school, gifted underachievers do not attend a Gymnasium as often as gifted achievers. For instance, only 61% of gifted underachievers (11 out of 18) in the Marburg Giftedness Project attended a Gymnasium. By contrast, the quota for the gifted achievers (who were carefully matched according to intelligence, gender, age, social background, and school environment) was 94% (16 out of 17) (Sparfeldt et al., 2006). Therefore, it is possible that gifted underachievers were underrepresented in our gifted sample. As gifted underachievers have a more negative motivational profile and lower self-esteem than gifted and non-gifted achievers (Hanses and Rost, 1998), it may well be the case that gifted underachievers also have lower life satisfaction. However, the percentage of gifted underachievers in representative samples is always quite low. In the Marburg Giftedness Project, only 18 students out of a total of 7023 (i.e., 12% of all gifted students) were identified as gifted underachievers (Rost and Hanses, 1997; Sparfeldt et al., 2006). Thus, the likelihood that our results are markedly distorted by an underrepresentation of gifted underachievers is quite low. Nevertheless, as our sample of gifted students was also rather small, future studies should try to replicate our results with larger samples that additionally comprise students from other school types, including gifted underachievers, to further enhance generalizability.

We used PSM to establish the comparability of the gifted and non-gifted groups. We used age, gender, and parental educational level as covariates to control for these potential nuisance variables. However, it would have been even better to include even more covariates, such as school environment (see Wirthwein and Rost, 2011). However, an analysis of a random sample drawn from all of the students we had at hand confirmed the results of the PSM.

We used only life satisfaction ratings. In future studies, it might be important to additionally focus on the affective components of subjective well-being (see Diener et al., 1999). Although there were no differences in positive or negative affect between gifted and non-gifted adults (Wirthwein and Rost, 2011), these components have yet to be investigated in gifted and non-gifted adolescents.

Despite some limitations of our study, we were able to show that there was on average no difference in life satisfaction in a vastly unselected gifted sample of adolescents as compared with non-gifted adolescents. Therefore, the disharmony hypothesis was not supported by our data. We were also able to disconfirm the hypothesis that gifted girls are especially prone to socio-emotional problems. Our findings go closely together with the main findings from the 29-year longitudinal Marburg Giftedness Study (Rost, 1993, 2009; Wirthwein and Rost, 2011) and other methodologically sound studies (see Plucker and Callahan, 2008): giftedness is not at all a risk factor for impaired psycho-social well-being or psycho-social development.

## Conflict of Interest Statement

The authors declare that the research was conducted in the absence of any commercial or financial relationships that could be construed as a potential conflict of interest.
